# Defect Engineering: Synthesis and Electrochemical Properties of Two‐Dimensional Mo_1.74_CT_*z*_ MXene

**DOI:** 10.1002/smsc.202400204

**Published:** 2024-08-07

**Authors:** Rodrigo M. Ronchi, Joseph Halim, Ningjun Chen, Per O. Å. Persson, Johanna Rosen

**Affiliations:** ^1^ Materials Design Division Department of Physics, Chemistry, and Biology (IFM) Linköping University SE‐581 83 Linköping Sweden; ^2^ Thin Film Physics Division Department of Physics, Chemistry, and Biology (IFM) Linköping University SE‐581 83 Linköping Sweden

**Keywords:** alloying, defects, electrochemical properties, MAX phase, MXene, pores, vacancies

## Abstract

The creation of vacancies and/or pores into two‐dimensional materials, like graphene and MXenes, has shown to increase their performance for sustainable applications. However, a simple and affordable method with controlled and tailorable vacancy concentration and/or pores size remains challenging. Herein, a simple and reproducible method is presented for controlled synthesis of Mo_1.74_CT_*z*_ MXene with randomly distributed vacancies and pores, obtained from selective etching of both Ga and Cr in the Cr‐alloyed MAX‐phase like precursor Mo_1.74_Cr_0.26_Ga_2_C. Structural and compositional analysis of the 3D alloy show ≈13% Cr on the metal site, homogeneously distributed between different particles and within the atomic structure. After etching, it translates to Mo_1.74_CT_*z*_ MXene, exhibiting defect‐rich sheets. Notably, the incorporation of Cr facilitates a shorter etching time with an improved yield compared to Mo_2_CT_*z*_. The Mo_1.74_CT_*z*_ MXene displays excellent electrochemical properties, almost doubling the capacitance values (1152 F cm^−3^ and 297 F g^−1^ at 2 mV s^−1^ scan rate), compared to its pristine counterpart Mo_2_CT_*z*_. The presented method and obtained results suggest defect engineering of MXenes through precursor alloying as a pathway that can be generalized to other phases, to further improve their properties for various applications.

## Graphical abstract

A simple and reproducible method for Mo_1.74_CT_*z*_ MXene synthesis with randomly distributed vacancies and pores is presented. The process allows shorter etching time, improved yield, and result in significantly higher capacitance compared to its stoichiometric MXene counterpart. Defect engineering through precursor alloying is suggested as a pathway to be generalized for further improvement of MXene properties.

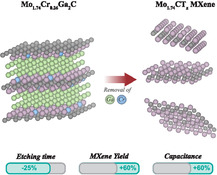

## 1 Introduction

Since the discovery of graphene,^[^ ^1^ ^]^ numerous two‐dimensional (2D) materials have been discovered and the research on these compounds expanded considerably due to their unique properties.^[^ ^2^ ^]^ Beyond graphene and its derivatives, 2D transition metal dichalcogenides, boron nitride, and phosphorene can be exfoliated from their weakly bonded van der Waals 3D parent phases.^[^ ^3^ ^]^ These materials are promising for numerous applications, including energy storage, water desalination and purification, and gas separation.^[^ ^4^, ^5^ ^]^

About a decade ago, the synthesis of MXenes raised the attention of the scientific community.^[^ ^6^ ^]^ MXenes are transition metal (M) carbides and/or nitrides (X) with the general formula M_*n*+1_X_*n*_T_*z*_, where *n* = 1–4 and T_*z*_ refers to surface terminations groups, originally in the form of O, –OH, and –F, though more recently being expanded to include elements such as Cl, Br, S, and Te.^[^ ^7^, ^8^ ^]^ They are traditionally produced by selective etching of the A element (such as Al, Si, and Ga) from the nanolaminated 3D MAX phase precursors.^[^ ^9^ ^]^ Since their discovery in 2011 (Ti_3_C_2_T_*z*_),^[^ ^6^ ^]^ more than 50 MXenes have been synthesized to date, and including the reported variations in surface terminations, this number increases significantly.^[^ ^9^ ^]^ Altogether, MXenes have showed promising results for a wide range of applications, including energy storage, catalysis, water purification, desalination, and in biomedicine.^[^ ^7^ ^]^

Environmental issues associated with the use of fossil fuels and rising energy consumption require further development and optimization of 2D materials to achieve high performance in sustainable applications. One potential route is to add vacancies and/or pores into the structure,^[^ ^4^ ^]^ which can play an essential role for many relevant properties. The introduction of pores in both graphene^[^ ^4^ ^]^ and MXenes^[^ ^10^ ^]^ has shown superior characteristics for molecular separation and electrochemical energy storage. For instance, introduction of ordered vacancies in Mo_4/3_CT_*z*_ and W_4/3_CT_*z*_ resulted in improved performance for supercapacitors and as an electrocatalyst for hydrogen evolution reaction (HER).^[^ ^11^, ^12^ ^]^

Several methods have been reported to create pores in MXenes. Mojtabavi et al. produced nanopores in Ti_3_C_2_T_*z*_ and Ti_2_CT_*z*_ using focused electron beam for DNA translocation and single‐molecule sensing applications.^[^ ^13^ ^]^ Another group used copper metal ions to catalyze and partially oxidize Ti_3_C_2_T_*z*_ sheets, resulting in TiO_2_ particles, which were then dissolved using HF, forming pores in the MXene sheets.^[^ ^10^ ^]^ Notably, an increased TiO_2_ content (compared to pristine material) was found by XPS, likely arising from oxidation facilitated by the Ti_3_C_2_T_*z*_ pores (more defective and largely exposed surfaces) and/or from not etched TiO_2_ from the pre‐oxidation procedure.^[^ ^10^ ^]^

Previously, we have shown randomly distributed clusters of vacancies in MXene flakes through selective etching of Sc atoms from the solid solution (Sc_0.33_Nb_0.67_)_2_AlC^[^ ^14^ ^]^ MAX phase. Ordered vacancy MXenes (Mo_4/3_CT_*z*_ and W_4/3_CT_*z*_) are representative compounds of an alternative way of forming vacancies with relatively good control of its concentration. This is a direct result from the use of quaternary in‐plane ordered MAX phases (*i‐*MAX) such as (Mo_2/3_M″_1/3_)_2_AlC and (W_2/3_M″_1/3_)_2_AlC, where M″ is Sc or Y^[^ ^15^, ^16^ ^]^ as starting materials. When exposed to hydrofluoric acid (HF), both Al and M″ elements are etched, resulting in vacancy ordering. However, the fixed ratio of metals in the MAX phase precursor does not allow tailoring of the fraction of vacancies. Therefore, controlled and tailored vacancy concentration and/or pore size remains challenging. Moreover, it is imperative to find a simple and straightforward method that could be applied to a broad range of MXenes.

Herein, we address this issue through the synthesis of Mo_2−*x*_CT_*z*_ MXene (*x* = 0.26) with defect engineering in the form of randomly distributed vacancy clusters. The synthesis is comprised of alloying 3D Mo_2_C with Cr, followed by a solid/liquid state reaction with Ga, forming Mo_2−*x*_Cr_*x*_Ga_2_C. The latter compound is a MAX phase‐like material according to standard definitions,^[^ ^17^ ^]^ but will here be referred to as a MAX phase. Subsequent HF‐etching and delamination procedures dissolve not only Ga, converting the MAX phase to 2D MXene, but also Cr, resulting in vacancies and pores randomly distributed in the structure. These results suggest a simple and reproducible method for creating vacancies and vacancy clusters in MXenes. Moreover, it could also allow tailoring of the vacancy fraction by changing the Cr content. The generation of defects in the 2D Mo_1.74_CT_*z*_ MXene resulted in excellent electrochemical properties, almost doubling the capacitance values compared to its pristine counterpart (Mo_2_CT_*z*_).

## 2 Results and Discussion

### 2.1 Alloying 3D Mo_2_C with Cr

Previous publications have indicated a wide range of Cr solubility in Mo_2_C carbide, with Cr contents up to around 60% around 1400 °C.^[^ ^18^ ^]^ However, based on the complete dissolution of Cr_2_AlC phase upon etching in HF,^[^ ^19^ ^]^ we have chosen to keep Mo as the majority element, since an expected high amount of defects generated by selective removal of a high concentration of Cr is likely to structurally disrupt the formation of the 2D MXene sheets. Initial synthesis attempts for a Cr concentration of 25% showed a low yield of the 3D MAX precursor, and thus 13% was chosen for the present study, further supported by previous work on Mo_2_C carbides alloyed with Cr by Knepfler and co‐workers.^[^ ^18^ ^]^ Notably, the present study targets a new method for defect engineering of Mo‐based MXenes and further experimental work is required to assess the maximum Cr concentration in the 221 MAX phase precursor.

From the results shown in Figure S1, Supporting Information, Cr was incorporated into the Mo_2_C structure, with an average concentration of (12 ± 1) %, which is consistent with the nominal powder ratios. The concentrations shown in Figure S1, Supporting Information, do not consider carbon because of the EDX uncertainties with respect to quantification of light elements. Assuming full occupancy of the C sites in Mo_2_C, we therefore refer to the sample as Mo_1.74_Cr_0.26_C. The addition of the smaller Cr atoms (atomic radii of Mo and Cr being 1.45 and 1.40 Å, respectively) does not change the space group, but decreases the lattice parameters from reported *a* = 4.74, *b* = 6.02, and *c* = 5.21 Å for pristine Mo_2_C^[^ ^20^, ^21^, ^22^ ^]^ to *a* = 4.70, *b* = 5.98, and *c* = 5.17 Å for Mo_1.74_Cr_0.26_C. It should be noted that although 3D Mo_2_C may occur in three different stable structures, we considered only the *Pbcn* space group in XRD and Rietveld analysis, since this is the reported most stable structure for temperatures below 1350 °C,^[^ ^21^, ^24^ ^]^ also supported by DFT calculations.^[^ ^23^, ^25^, ^26^ ^]^ Notably, >99% phase purity was achieved.

### 2.2 Synthesis of 3D Mo_2_Ga_2_C Alloyed with Cr

**Figure 1:**
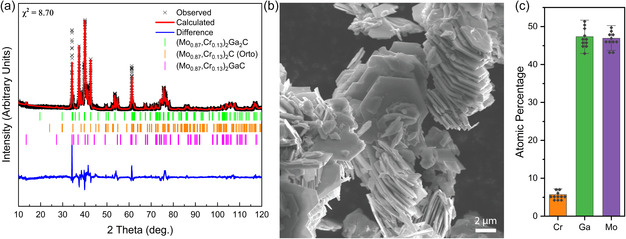
a) Rietveld analysis of the MAX phase sample: The black “X” represents the measured pattern, with the Rietveld generated pattern in red and the difference between them in blue. The ticks below the pattern represent the peak positions of Mo_2−*x*_Cr_*x*_Ga_2_C (green), orthorhombic Mo_2−*x*_Cr_*x*_C (orange), and Mo_2−*x*_Cr_*x*_GaC (pink). b) SEM micrograph showing the lamellar morphology. c) Chemical composition obtained through EDX measurements on 15 individual 221 MAX phase particles.

Rietveld analysis of the synthesized Mo_2−*x*_Cr_*x*_Ga_2_C is shown in **Figure** **1**a (further details in Table S2, Supporting Information). Three phases are identified: Mo_2−*x*_Cr_*x*_Ga_2_C (targeted phase, 42 wt%) Mo_2−*x*_Cr_*x*_GaC and Mo_2−*x*_Cr_*x*_C. Mo_2_Ga_2_C, Mo_2_GaC and Cr_2_GaC phases have been synthesized in both thin film and powder forms.^[^ ^16^, ^17^, ^27^, ^28^, ^29^ ^]^ However, the Cr_2_Ga_2_C phase has never been reported, not even as an impurity phase. It is therefore likely that the Cr_2_Ga_2_C phase is not stable and thus, the more Cr is added during synthesis, less stable 221 phase becomes. The lattice parameters for the Mo_2−*x*_Cr_*x*_Ga_2_C alloy (*a* = 3.027 and *c* = 18.032 Å) are smaller compared with the pure Mo_2_Ga_2_C (*a* = 3.064 and *c* = 18.153 Å),^[^ ^30^ ^]^ qualitatively confirming the presence of the smaller Cr atoms in the structure.

The SEM image (Figure 1b) shows the typical morphology of a laminated MAX phase‐like material which is similar to previous results for Mo_2_Ga_2_C.^[^ ^27^ ^]^ The average atomic percentages obtained from EDX, shown in Figure 1c, were (6 ± 1) for Cr, (47 ± 3) for Ga, and (47 ± 2) % for Mo, which is again consistent with the nominal Mo/Cr ratios and the targeted Ga content. From the assumption of full occupancy of the C sites, we therefore refer to the sample as Mo_1.74_Cr_0.26_Ga_2_C. Importantly, there is no sign of Cr being selectively lost to other impurity phases, which is critical for the control of the defects in the subsequent MXene synthesis.

**Figure 2:**
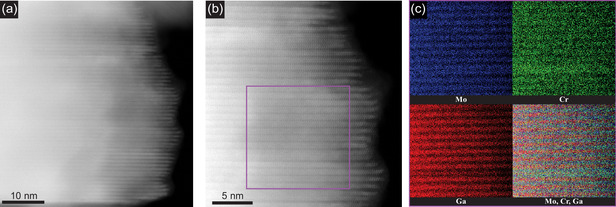
a,b) STEM images of Mo_1.74_Cr_0.26_Ga_2_C under different magnifications. c) EDX of selected area shown in (b).

While no significant deviation in particle composition was found in the SEM‐EDX analysis, high resolution microscopy was performed to identify potential Cr/Mo clustering within the alloy. STEM images of the MAX phase are shown in **Figure** **2**a,b, in which the layered atomic structure is clearly seen. The structure can be identified as primarily a 221 MAX phase with a small amount of 211 phase displayed as a few intergrown layers in the 221 matrix. EDX maps (Figure 2c) further show the coexistence of both Mo_2−*x*_Cr_*x*_Ga_2_C and Mo_2−*x*_Cr_*x*_GaC, as previously seen in the Rietveld analysis. There is also an indication of a slightly higher amount of Cr in the Mo_2−*x*_Cr_*x*_GaC phase. More importantly, the EDX maps do not show any sign of Cr agglomeration within any of the 221 and 211 MAX phases, with Mo and Cr atoms being uniformly distributed. In addition, the Cr atoms are uniformly distributed within the Mo layers only, indicating the substitution of Mo atoms by Cr (also corroborated by a decrease in the Mo content upon Cr addition, as shown in Figure 1c).

### 2.3 MXene Synthesis

**Figure 3:**
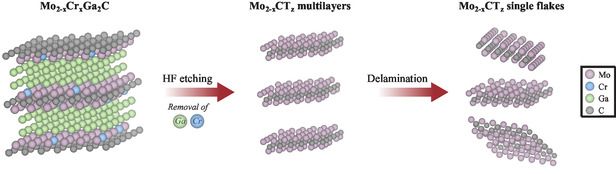
Schematic of the MXene synthesis procedure, comprising etching of the MAX phase alloy Mo_2−*x*_Cr_*x*_Ga_2_C, with intentional vacancy (defect) generation from Cr removal, and delamination of the resulting multilayer MXene into single sheets.

A schematic of the etching procedure used is presented in **Figure** **3**. Etching Mo_1.74_Cr_0.26_Ga_2_C MAX phase in HF selectively removes both the Ga and the Cr atoms and produces multilayer Mo_2−*x*_CT_*z*_ MXene with randomly distributed vacancies. The MXene is subsequently delaminated into single sheets by intercalation with TBAOH.

**Figure 4:**
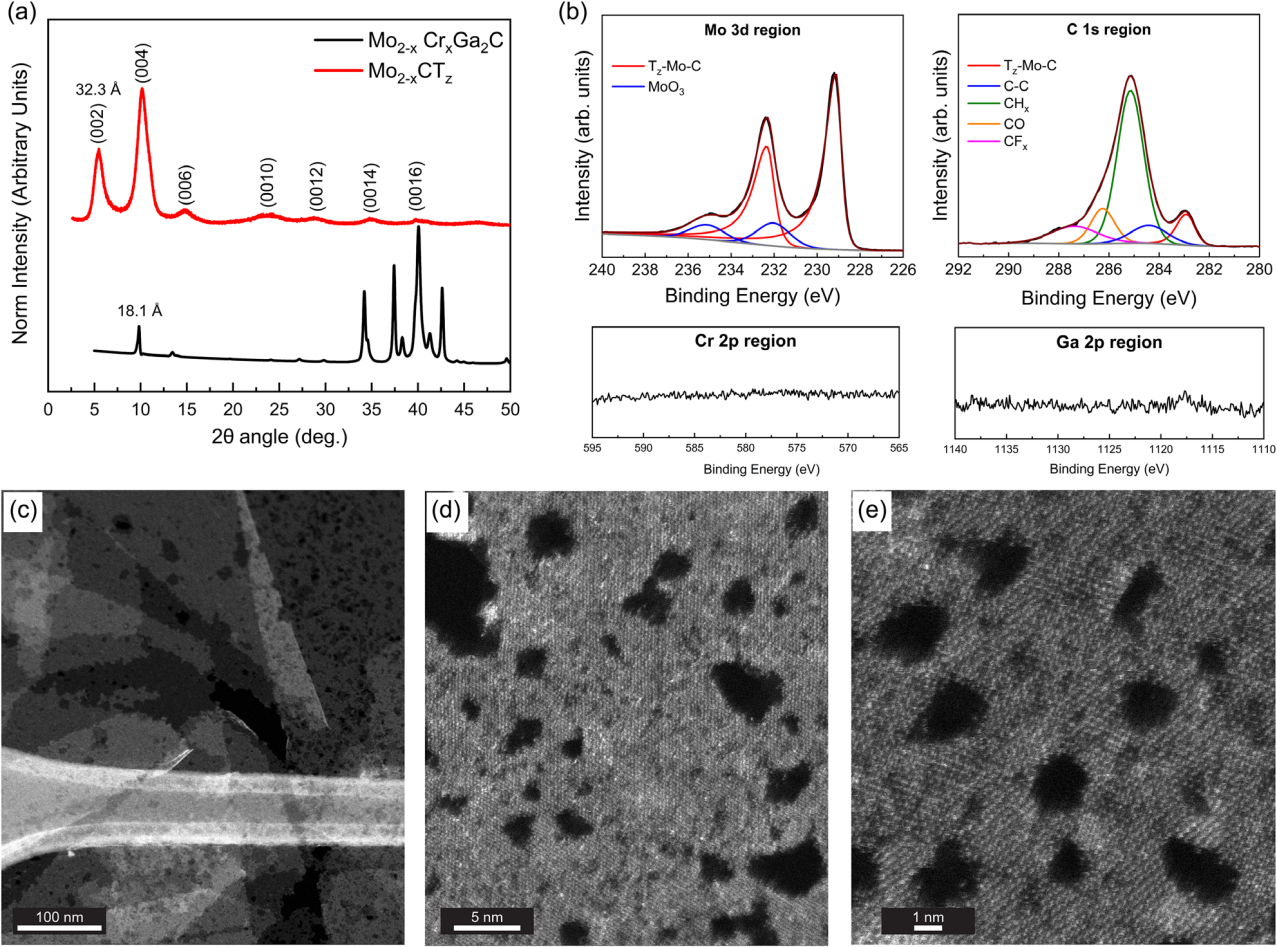
a) XRD data for the 3D Mo_2−*x*_Cr_*x*_Ga_2_C powder sample and a filtered free‐standing film of Mo_2−*x*_CT_*z*_ MXene. The values of the (002) peaks represent the double interlayer spacing. b) XPS spectra of the Mo_2−*x*_CT_*z*_ film for the Mo 3*d*, C 1*s*, Cr 2*p*, and Ga 2*p* regions, respectively. The color‐coded peak fittings represent the assigned species tabulated in the Supporting Information. c) Multiple overlapping single MXene sheets with well‐defined outlines. Some sheets reveal many small pores while others display fewer number of defects but with larger holes. d) High magnification image showing the range of pore distributions, from near single atom sized pores adjacent to significantly larger pores. e) Lattice resolved image to verify the preservation of the hexagonal structure.

The XRD pattern of a Mo_2−*x*_CT_*z*_ free standing film (**Figure** **4**a) shows the typical shift of the (002) peak upon conversion of the 3D MAX phase into 2D MXene, increasing the interlayer spacing *d*_c/2_ from ≈9 to 16 Å (for the MAX phase, it corresponds to c‐LP). The increase originates from replacement of Al atoms with surface termination species –O, –OH, and –F, and intercalation of water molecules between the layers.^[^ ^27^ ^]^

The MAX to MXene conversion yield was calculated by comparing the mass of 2D material obtained after etching and delamination and the amount of its respective 3D phase present in the initial powder. Note that in a multiphase sample, the amount of MAX phase precursor was estimated by Rietveld refinement, and terminations of the MXene were not included in the molar mass. Since both Ga and Cr are selectively etched (see analysis below), 1 g of Mo_1.74_Cr_0.26_Ga_2_C can provide a maximum of 0.54 g of Mo_1.74_CT_*z*_ MXene (in the case of 100% conversion) and this normalization needs to be taken into account. From two batches, the calculated average MAX to MXene conversion yield was 16%, similar to previously reported 16% for (Mo_2/3_Y_1/3_)_2_AlC *i*‐MAX,^[^ ^31^ ^]^ and slightly higher than previously shown 10% for Mo_2_Ga_2_C.^[^ ^27^ ^]^

High‐resolution XPS spectra with peak fitting are shown for a Mo_2−*x*_CT_*z*_ filtered film in Figures 4b and S2, Supporting Information (surface termination species). The corresponding compositions extracted from the fitting can be found in (Table S3 and S4, Supporting Information). The differences in binding energies (BE) of T_*z*_‐Mo‐C when comparing Mo 3*d* and C 1*s* regions of Mo_2−*x*_CT_*z*_ with respective species in both Mo_4/3_CT_*z*_ *i*‐MXene (produced from (Mo_2/3_Sc_1/3_)_2_AlC) and Mo_2_CT_*z*_ MXene (produced from Mo_2_Ga_2_C)^[^ ^32^ ^]^ is small, within ±0.2 eV. The chemical formula of Mo_2−*x*_CT_*z*_ extracted from the atomic percentages is Mo_1.74 ± 0.06_CO_0.95 ± 0.02_(OH)_0.63 ± 0.01_F_0.3 ± 0.03_.0.2 ± 0.05H_2_O_ads_, though it should be noted that the Mo faction can be reduced with up to 0.3 without changing the quality (error percentage) of the fit. While we do not expect a drastic change in the Mo:C ratio after etching, we cannot exclude such change based on the XPS analysis. The total sum of the terminations (1.88) is close to the ideal value of 2, which is similar to what was reported for Mo_2_CT_*z*_^[^ ^32^ ^]^ (1.75). More importantly, the Cr 2*p* and Ga 2*p* regions do not show any peak signal, indicating that both Cr and Ga have been completely etched upon MAX to MXene conversion.

The removal of both the A‐layer element (e.g., Al), and an element from the M‐layers (e.g., Y and Sc), has been shown previously for both ordered^[^ ^15^, ^31^ ^]^ and disordered^[^ ^14^ ^]^ distributions of two *M*‐elements in a MAX phase. The preferred etching of Cr (compared to Mo) is most likely due to a higher reactivity of Cr in an HF solution. Such behaviour has been suggested in a theoretical study on MAX to MXene conversion and through experiments,^[^ ^19^ ^]^ where it was indicated that Cr_2_AlC MAX phase is prone to have both Al and Cr elements etched and, hence, is not likely to form a MXene (the MAX phase dissolves completely). The results obtained in the present study also corroborates what has been found for other Mo‐based MAX phases, such as Mo_2_Ga_2_C, Mo_2_TiAlC_2_, Mo_2_Ti_2_AlC_3_, in which only the Al layer (not Mo) is removed upon etching.

In order to have a better understanding of how the etching of Cr atoms affects the MXene structure, STEM analysis was carried out (Figure 4c). Large (>1 μm) single sheets are clearly visible. Unlike over‐etched fragmented MXene sheets which typically show fractal edges, these ones maintain well‐defined sheets with clearly outlined edges. However, each sheet contains multiple holes with a typical diameter of 1–10 nm (see Figure 4d,e). Occasionally, these holes form chains, resulting in “scratches” that stretch over several hundred nm (not shown). The density and size distribution of the holes varies, meaning that some sheets can contain a higher density of very small holes, while others may have a lower number of larger holes, resulting in a reduced sheet surface area. Nonetheless, as shown in Figure 4e, the MXene structure is still preserved, although individual and clusters of atomic defects can be observed, in line with previous reports.^[^ ^33^, ^34^ ^]^

Since the MAX phase did not show any signs of Cr agglomeration (Figure 2) the presence of holes in the MXene sheet is intriguing. Previously, (S)TEM evaluation of ordered vacancy MXenes Mo_4/3_CT_*z*_^[^ ^15^ ^]^ showed defects in the form of single vacancies, whilst Nb_1.33_CT_*z*_ Mxene with randomly disordered vacancies had vacancy clusters up to 4 nm.^[^ ^14^ ^]^ This indicates that selective removal of Cr atoms in the present work may reduce the stability of the surrounding structure, making it more prone to be dissolved in the acidic environment. Such destabilizing effect was previously observed in the etching of (Mo_*x*_Sc_1−*x*_)_2_AlC *i*‐MAX phase.^[^ ^35^ ^]^ Although *i*‐MAX phase was formed for different metal concentrations, Mo_4/3_CT_*z*_ MXene sheets were obtained for *x* = 2/3, while no MXene could be synthesized with higher Sc concentrations, i.e., *x* = 1/2 or 1/3 (the phase stability was reduced and the MXene dissolved upon attempted etching). Based on these observations, the synthesis conditions can likely be optimized to further control the structural quality of the 2D material. Although the etching conditions used here (25 HF%, 120 h at RT) are milder and shorter compared to previously used etching conditions for pure Mo_2_Ga_2_C (25 HF%, 160 h at 55 °C),^[^ ^27^, ^32^ ^]^ even milder etching conditions may be possible, such as shortening the time or even using other etchants such as LiF‐HCl (48 h at RT for Mo_2_Ga_2_C).^[^ ^27^ ^]^

### 2.4 Electrochemical Characterization

**Figure 5:**
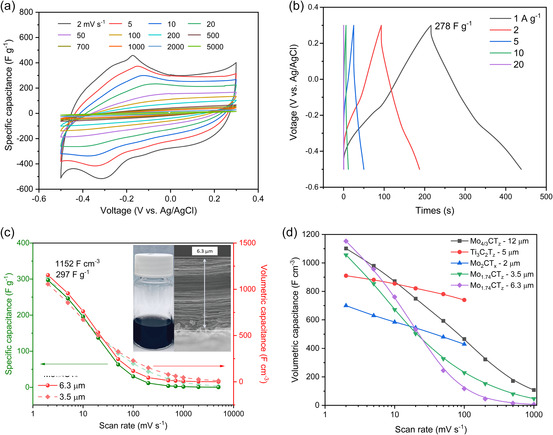
Electrochemical performance: a) CV data for a 6.3 μm thick Mo_1.74_CT_*z*_ MXene film, collected at scan rates from 2 to 1000 mV s^−1^, b) GCD curves at different current density, and c) volumetric and gravimetric capacitance at different scan rates. Inset: Liquid suspension of delaminated Mo_1.74_CT_*z*_ MXene in water and a SEM micrograph of the cross section of the filtered film. d) Comparison of the volumetric capacitance of the present work with previously reported values.

The Mo_1.74_CT_*z*_ MXene was tested as an electrode and assessed in 1 m H_2_SO_4_ electrolyte. The CV curves in **Figure** **5**a show a typical pseudocapacitive behaviour with distinct redox peaks in a potential window from −0.5 to 0.3 V. Figure 5b shows the nearly symmetrical triangular GCD curves during charge and discharge for different current densities, demonstrating an excellent capacitive performance as well as rapid and reversible faradaic reactions of the MXene electrode. The maximum volumetric and gravimetric capacitance values of 1152 F cm^−3^ and 297 F g^−1^ were achieved at 2 mV s^−1^, respectively (Figure 5c). Notably, the obtained volumetric capacitance is comparable to the one previously reported for Mo_4/3_CT_*z*_ (1153 F cm^−3^) and well above reported values for Ti_3_C_2_T_*z*_ (900 F cm^−3^)^[^ ^36^ ^]^ and Mo_2_CT_*z*_ (700 F cm^−3^)^[^ ^27^ ^]^ for the same scan rates, as can be seen in Figure 5d. Both the vacancy ordered Mo_4/3_CT_*z*_ MXene and the here presented Mo_1.74_CT_*z*_ MXene with disordered vacancies/pores display very similar capacitance curves, with exceptional values at low scan rates, though with decreasing capacitance for higher scan rates. This behaviour contrasts with non‐vacancy MXenes, like Ti_3_C_2_T_*z*_ or Mo_2_CT_*z*_, which shows a better rate capability with a less rapid decrease in capacitance for higher scan rates. Thus, the defects in Mo_1.74_CT_*z*_, or a change in surface chemistry originating from the defect formation, seems to enhance the capacitance considerably (almost doubling the values shown for Mo_2_CT_*z*_), but the rate capability requires further attention.

The Mo_1.74_CT_*z*_ MXene electrode shows excellent stability, with a capacitance retention of 79% after 10.000 charge/discharge cycles (see Figure S3a, Supporting Information). It should be noted that the coulombic efficiency is around 100% even after 10.000 cycles, further demonstrating the good electrochemical performance. In addition, the Nyquist plots in Figure S3b, Supporting Information, display a lower charge transfer resistance and ion transport resistance in the mid‐to‐high frequency region, and the imaginary part of the impedance is nearly vertical to the real part in the low‐ frequency region, which indicates an ideal capacitive behaviour.

Altogether, the present study shows defect engineering as a viable path for improving MXene performance. The changed properties arise from the formation of vacancies and holes (pores) in the MXene sheet, which, in turn, originates from the selective etching of Ga as well as Cr from the 3D precursor alloy. We suggest that the incorporation of Cr increases the reactivity of the precursor, evident from a reduced etching time as well as an improved yield compared to the pure ternary counterpart, Mo_2_Ga_2_C. We also suggest that the same approach may be used for other MAX phases, i.e., alloying with an element (here Cr) to make the material more susceptible to etching. The control of generated defects and morphology expands the property space of MXenes, increasing their range of potential applications.

## 3 Conclusions

A simple, facile, and reproducible method for producing random vacancies and holes (pores) in Mo‐based MXenes is here presented. The defect engineering is facilitated by alloying the 3D precursor material with Cr, through a two step‐procedure involving the synthesis of both Mo_2−*x*_Cr_*x*_C and Mo_2−*x*_Cr_*x*_Ga_2_C (*x* = 0.26), after which both the Cr (metal) and Ga (A‐group) elements present in the quaternary MAX phase solid‐solution are selectively etched. The synthesized 2D Mo_1.74_CT_*z*_ MXenes had disordered vacancies and pores in a process that required a shorter etching time for an improved yield compared to Mo_2_CT_*z*_. The structural defects provided less dense flakes, which may be beneficial for various storage and conversion technologies. Initial tests of electrochemical energy storage capabilities show volumetric and gravimetric capacitances of 1152 F cm^−3^ and 297 F g^−1^ at 2 mV s^−1^ scan rate, respectively, with a capacitance retention of 79.1% after 10.000 charge/discharge cycles. This is superior to other pristine MXenes without additives, including stoichiometric Mo_2_CT_*z*_ MXene. The presented method and obtained results suggest defect engineering as a pathway for further improvement of MXene properties for various applications.

## 4 Experimental Section

4.1

4.1.1

##### Materials Synthesis

The 3D Mo_2−*x*_Cr_*x*_C powder was synthesized via a solid‐state reaction using elemental powders as precursors. Using mortar and pestle, Mo (99.95 wt%, 3–7 μm, Alfa Aesar), Cr (>99 wt%, <325 mesh, Sigma Aldrich), and C (99.9995 wt%, 200 mesh, Alfa Aesar) powders were mixed in a molar ratio 1.74: 0.26: 1, respectively, i.e., a nominal Cr metal concentration of 13%. The powder mixture was placed in an alumina crucible and heated in a horizontal alumina tube furnace at 1400 °C for 25 h under a 5 sccm Ar flow (heating and cooling rates were 5 °C min^−1^). The obtained lightly sintered sample was crushed using mortar and pestle and then sieved through a 36 μm sieve.

The 3D Mo_2−*x*_Cr_*x*_Ga_2_C was obtained using a solid/liquid reaction between the previously synthesized Mo_2−*x*_Cr_*x*_C powder and Ga (rods, 99.999 wt%, Wuhan Xinrong Bew Material Co. Ltd., Wuhan, China), in a molar ratio 1:8, respectively, i.e., with excess Ga compared to the targeted phase. A hot water bath was used to melt the Ga rods, and a total mass of 10 g of Ga droplets (from a pipette) and Mo_2−*x*_Cr_*x*_C powders were added to an alumina crucible in alternating layers, and the material was mixed thoroughly. The crucible was inserted in a horizontal quartz tube furnace and heated up to 740 °C for 6.5 days (under a 5 sccm Ar flow), with heating and cooling rates of 10 °C min^−1^. The obtained sample was added to a 6 m hydrochloric acid solution (12 m, Fisher Chemicals, Germany) to remove the excess Ga. Due to the strongly exothermal reaction, about 1 g of the sample was added every 5 min to avoid overheating. The mixture was stirred overnight using a magnetic stirrer and was subsequently washed with deionized water (DI) to achieve pH ≈ 6. The washing cycles were composed of centrifuge sedimentation (6000 rpm, 4427 rcf, for 2 min) and decanting the supernatant several times. Finally, the washed sample was filtered through a nanoporous polypropylene membrane (3501 coated PP, 0.064 μm pore size, Celgard, LLC, USA) using vacuum‐assisted filtration, crushed using mortar and pestle, and then sieved through a 36 μm sieve.

Multilayered Mo_2−*x*_CT_*z*_ MXene was synthesized by selective etching of the Mo_2−*x*_Cr_*x*_Ga_2_C solid solution. Three grams of the quarternary precursor were mixed with 50 mL of 25 vol% hydrofluoric acid (HF, 50 vol%, Sigma Aldrich AB, Stockholm) in a PTFE bottle. The mixture was magnetically stirred at room temperature for 5 days, after which the mixture was washed with DI water through several cycles until reaching pH ≈ 6. Delamination of the MXene multilayers were performed in line with previous reported procedures.^[^ ^37^ ^]^ 30 mL of 25 vol% tetrabutylammonium hydroxide (TBAOH, Sigma Aldrich, AB, Stockholm, Sweden) was added to the sedimented powder in a centrifuge tube. The mixture was shaken for 5 min in a vortex mixer (LSE, Corning Inc., Glendale, AZ) at a speed of 1000 rpm, 162 rcf, and then centrifugated (6000 rpm, 4427 rcf, for 1 min) prior to TBAOH decantation. The sedimented powder was then washed three times with 40 mL of N_2_‐deaerated DI water to remove all TBAOH. Care was taken to keep the tube stationary during the DI water pouring procedure to avoid spontaneous delamination of the MXene. For the final delamination step, 40 mL of N_2_‐deaerated DI water was added to the sedimented MXene and was then shaken for 15 min using a vortex mixer followed by centrifugation (5400 rpm, 3528 rcf, for 1 h). The remaining single or few‐layer flakes of MXene present in the supernatant were collected. Finally, the resulting suspension (concentration = 1.57 mg mL^−1^) was vacuum filtered on a nanoporous polypropylene membrane (3501 Coated PP, 0.064 μm pore size, Celgard, LLC, USA) in air, resulting in free‐standing films.

##### Materials Characterization

X‐ray diffraction (PANalytical diffractometer, Cu K_α_ radiation source) was employed to evaluate the crystal structure. The divergence and receiving slits used were 1/2° and 5 mm and a nickel filter was added to avoid any *K*_β_ signal. The step size and time per step were 0.0084° and 32 s, respectively. A Rietveld refinement procedure^[^ ^38^, ^39^ ^]^ was used to obtain structural parameters and weight percentages of the different phases present in the sample (carried out using FULLPROF software^[^ ^40^ ^]^). The peak shapes were modeled using Thompson‐Cox‐Hastings pseudo‐Voigt with Axial divergence asymmetry function, in which the instrument parameters were determined from a NIST LaB_6_ standard. The refined parameters were; scale factors, zero offset, five background parameters (of a polynomial function), lattice parameters (LPs), Y‐parameter, asymmetry parameters, overall thermal factor, and atomic positions. Motivated by performed materials composition analysis (see below), Mo/Cr occupancy was not refined.

The morphology and chemical composition of the 3D powders were analysed by scanning electron microscopy, SEM (LEO 1550), combined with an energy dispersive X‐ray (EDX) spectrometer. The EDX measurements were acquired from at least 15 particles for every sample.

To investigate the atomic structure and defects, high‐angle annular dark‐field (HAADF) scanning transmission electron microscopy (HAADF‐STEM) imaging and EDX elemental analysis were performed using the Linköping double‐corrected FEI Titan^3^ (S)TEM electron microscope operated at 300 kV. Samples for STEM were prepared by drop casting the as‐prepared powders or the MXene solution onto lacey carbon TEM grids that were vacuum dried prior to analysis.

X‐ray photoelectron spectroscopy (XPS) measurements were performed on filtered free‐standing MXene films, attached to the holder using a double‐sided carbon tape and copper contacts to ensure that the sample was grounded. The surface analysis system (Kratos AXIS UltraDLD, Manchester, UK) is comprised of monochromatic Al‐K_α_ (1486.6 eV) radiation with a 45° angle between the sample surface and the X‐ray beam (spot size ≈300 × 800 μm). Charge neutralization was performed using a co‐axial, low energy (≈0.1 eV) electron flood source to avoid shifts in the recorded binding energy (BE). XPS spectra were recorded for F 1*s*, O 1*s*, C 1*s*, Ga 2*p*, Mo 3*d*, and Cr 2*p*. The analyzer's pass energy used for all the regions was 20 eV with a step size of 0.1 eV. The BE scale of all XPS spectra was referenced to the Fermi‐edge (*E*_F_), which was set to a BE of 0 (zero) eV. The peak fitting was carried out using CasaXPS^[^ ^41^ ^]^ Version 2.3.16 RP 1.6, and the fitting and quantification of the global elemental percentages were performed in the same manner as in previous work.^[^ ^27^, ^32^, ^42^ ^]^

The electrochemical evaluation (potentiostat Biological VSP), data collected using glassy carbon current collectors, was performed in a three‐electrode Swagelok cell in 1 m H_2_SO_4_ electrolyte. The MXene‐free standing film was the working electrode, activated carbon (PTFE 10 wt%) was used as the counter electrode and Ag/AgCl as the reference electrode. The density used to calculate the volumetric capacitance of the MXene film was 3.88 g cm^−3^. The cyclic voltammetry (CV) and galvanic charge discharge (GCD) curves were recorded at different scan rates and current densities, respectively. The electrochemical impedance spectroscopy (EIS) data was collected under open‐circuit potential in the frequency range from 0.01 to 105 Hz. Finally, the cyclic stability was measured at a current density of 10 A g^−1^.

## Conflict of Interest

The authors declare no conflict of interest.

## Supporting information

Supplementary Material

## Data Availability

The data that support the findings of this study are available from the corresponding author upon reasonable request.

## References

[1] K. S. Novoselov , A. K. Geim , S. V. Morozov , D. Jiang , Y. Zhang , S. V. Dubonos , I. V. Grigorieva , A. A. Firsov , Science 2004, 306, 666. 15499015 10.1126/science.1102896

[2] V. Nicolosi , M. Chhowalla , M. G. Kanatzidis , M. S. Strano , J. N. Coleman , Science 2013, 340, 1226419.

[3] K. S. Novoselov , A. Mishchenko , A. Carvalho , A. H. Castro Neto , Science 2016, 353, aac9439. 27471306 10.1126/science.aac9439

[4] T. Yang , H. Lin , X. Zheng , K. P. Loh , B. Jia , J. Mater. Chem. A 2017, 5, 16537.

[5] J. Pang , R. G. Mendes , A. Bachmatiuk , L. Zhao , H. Q. Ta , T. Gemming , H. Liu , Z. Liu , M. H. Rummeli , Chem. Soc. Rev. 2018, 48, 72. 10.1039/c8cs00324f 30387794

[6] M. Naguib , M. Kurtoglu , V. Presser , J. Lu , J. Niu , M. Heon , L. Hultman , Y. Gogotsi , M. W. Barsoum , Adv. Mater. 2011, 23, 4248. 21861270 10.1002/adma.201102306

[7] A. VahidMohammadi , J. Rosen , Y. Gogotsi , Science 2021, 372, eabf1581. 34112665 10.1126/science.abf1581

[8] V. Kamysbayev , A. S. Filatov , H. Hu , X. Rui , F. Lagunas , D. Wang , R. F. Klie , D. V. Talapin , Science 2020, 369, 979. 32616671 10.1126/science.aba8311

[9] J. Zhou , M. Dahlqvist , J. Björk , J. Rosen , Chem. Rev. 2023, 123, 13291. 37976459 10.1021/acs.chemrev.3c00241 PMC10722466

[10] C. E. Ren , M. Q. Zhao , T. Makaryan , J. Halim , M. Boota , S. Kota , B. Anasori , M. W. Barsoum , Y. Gogotsi , ChemElectroChem 2016, 3, 689.

[11] B. Ahmed , A. El Ghazaly , J. Rosen , Adv. Funct. Mater. 2020, 30, 2000894.

[12] H. Lind , B. Wickman , J. Halim , G. Montserrat‐Sisó , A. Hellman , J. Rosen , Adv. Sustainable Syst. 2020, 5, 2000158.

[13] M. Mojtabavi , A. Vahidmohammadi , W. Liang , M. Beidaghi , M. Wanunu , ACS Nano 2019, 13, 3042. 30844249 10.1021/acsnano.8b08017

[14] J. Halim , J. Palisaitis , J. Lu , J. Thörnberg , E. J. Moon , M. Precner , P. Eklund , P. O. A. Persson , M. W. Barsoum , J. Rosen , ACS Appl. Nano Mater. 2018, 1, 2455.

[15] Q. Tao , M. Dahlqvist , J. Lu , S. Kota , R. Meshkian , J. Halim , J. Palisaitis , L. Hultman , M. W. Barsoum , P. O. Å. Persson , J. Rosen , Nat. Commun. 2017, 8, 14949. 28440271 10.1038/ncomms14949 PMC5413966

[16] R. Meshkian , M. Dahlqvist , J. Lu , B. Wickman , J. Halim , J. Thörnberg , Q. Tao , S. Li , S. Intikhab , J. Snyder , M. W. Barsoum , M. Yildizhan , J. Palisaitis , L. Hultman , P. O. Ä. Persson , J. Rosen , Adv. Mater. 2018, 30, 1706409. 10.1002/adma.201706409 29633399

[17] M. Dahlqvist , M. W. Barsoum , J. Rosen , Mater. Today 2024, 72, 1.

[18] C. A. Knepfler , K. T. Faber , J. Weertman , G. B. Olson , C. R. Hubbardb , O. B. Cavin , N. Packen , J. Alloys Compd. 1997, 248, 139.

[19] J. Björk , J. Halim , J. Zhou , J. Rosen , npj 2D Mater. Appl. 2023, 7, 1.

[20] J. Dubois , T. Epicier , C. Esnouf , G. Fantozzi , Acta Metall. 1988, 36, 1891.

[21] T. Epicier , J. Dubois , C. Esnouf , G. Fantozzi , P. Convert , Acta Metall. 1988, 36, 1903.

[22] K. M. Reddy , T. N. Rao , J. Revathi , J. Joardar , J. Alloys Compd. 2010, 494, 386.

[23] H. Liu , J. Zhu , Z. Lai , R. Zhao , D. He , Scr. Mater. 2009, 60, 949.

[24] E. Parthé , V. Sadogopan , Acta Crystallogr. 1963, 16, 202.

[25] Y. Liu , Y. Jiang , R. Zhou , X. Liu , J. Feng , Ceram. Int. 2015, 41, 5239.

[26] C. De Oliveira , D. R. Salahub , H. A. De Abreu , H. A. Duarte , J. Phys. Chem. C 2014, 118, 25517.

[27] J. Halim , S. Kota , M. R. Lukatskaya , M. Naguib , M. Q. Zhao , E. J. Moon , J. Pitock , J. Nanda , S. J. May , Y. Gogotsi , M. W. Barsoum , Adv. Funct. Mater. 2016, 26, 3118.

[28] J. Etzkorn , M. Ade , D. Kotzott , M. Kleczek , H. Hillebrecht , J. Solid State Chem. 2009, 182, 995.

[29] A. Petruhins , A. S. Ingason , M. Dahlqvist , A. Mockute , M. Junaid , J. Birch , J. Lu , L. Hultman , P. O. A. Persson , J. Rosen , Phys. Status Solidi RRL 2013, 7, 971.

[30] C. C. Lai , R. Meshkian , M. Dahlqvist , J. Lu , L. Näslund , O. Rivin , E. N. Caspi , O. Ozeri , L. Hultman , P. Eklund , M. W. Barsoum , J. Rosen , Acta Mater. 2015, 99, 157.

[31] J. Halim , A. S. Etman , A. Elsukova , P. Polcik , J. Palisaitis , M. W. Barsoum , P. O. Å. Persson , J. Rosen , Nanoscale 2021, 13, 311. 33338088 10.1039/d0nr07045a

[32] W. Zheng , J. Halim , P. O. Å. Persson , J. Rosen , M. W. Barsoum , J. Power Sources 2022, 525, 231064.

[33] L. H. Karlsson , J. Birch , J. Halim , M. W. Barsoum , P. O. Ä. Persson , Nano Lett. 2015, 15, 4955. 26177010 10.1021/acs.nanolett.5b00737

[34] X. Sang , Y. Xie , D. E. Yilmaz , R. Lot , M. Alhabeb , A. Ostadhossein , B. Anasori , W. Sun , X. Li , K. Xiao , P. R. C. Kent , A. C. T. Van Duin , Y. Gogotsi , R. R. Unocic , Nat. Commun. 2018, 9, 2266/1. 29891836 10.1038/s41467-018-04610-0 PMC5995835

[35] A. Mockute , Q. Tao , M. Dahlqvist , J. Lu , S. Calder , E. N. Caspi , L. Hultman , J. Rosen , Phys. Rev. Mater. 2019, 3, 113607.

[36] M. Ghidiu , M. R. Lukatskaya , M.‐Q. Zhao , Y. Gogotsi , M. W. Barsoum , Nature 2014, 516, 78. 25470044 10.1038/nature13970

[37] M. Naguib , R. R. Unocic , B. L. Armstrong , J. Nanda , Dalton Trans. 2015, 44, 9353. 25912071 10.1039/c5dt01247c

[38] R. A. Young , in The Rietveld Method, 1st ed. (Ed: R. A. Young ), Oxford University Press, Oxford 1993.

[39] H. M. Rietveld , J. Appl. Crystallogr. 1969, 2, 65.

[40] J. Rodríguez‐Carvajal , Phys. B: Condens. Matter 1993, 192, 55.

[41] N. Fairley , V. Fernandez , M. Richard‐plouet , C. Guillot‐deudon , J. Walton , E. Smith , D. Flahaut , M. Greiner , M. Biesinger , S. Tougaard , D. Morgan , J. Baltrusaitis , Appl. Surf. Sci. Adv. 2021, 5, 100112.

[42] H. Lind , J. Halim , S. I. Simak , J. Rosen , Phys. Rev. Mater. 2017, 1, 044002.

