# Supplementary material for: Defect Engineering: Synthesis and Electrochemical Properties of Two‐Dimensional Mo1.74CT z MXene
Source: Small Sci. 2024 Aug 7;4(10):2400204. doi: 10.1002/smsc.202400204 (PMC11935066; doi:10.1002/smsc.202400204)
Supplement: Supplementary file 1 — Supplementary Material [file SMSC-4-2400204-s001.pdf]

Defect engineering: Synthesis and electrochemical properties of two-dimensional $Mo_{1.74}CT_z$ MXene.

Rodrigo M. Ronchi [1], Joseph Halim *[1], Ningjun Chen [1], Per O. Å. Persson [2], Johanna Rosen *[1]

[1] Materials Design division, Department of Physics, Chemistry, and Biology (IFM), Linköping University, SE-581 83 Linköping, Sweden.

[2] Thin Film Physics division, Department of Physics, Chemistry, and Biology (IFM), Linköping University, SE-581 83 Linköping, Sweden.

*Corresponding authors: joseph.halim@liu.se, johanna.rosen@liu.se

3D $Mo_{2-x}Cr_xC$ POWDER

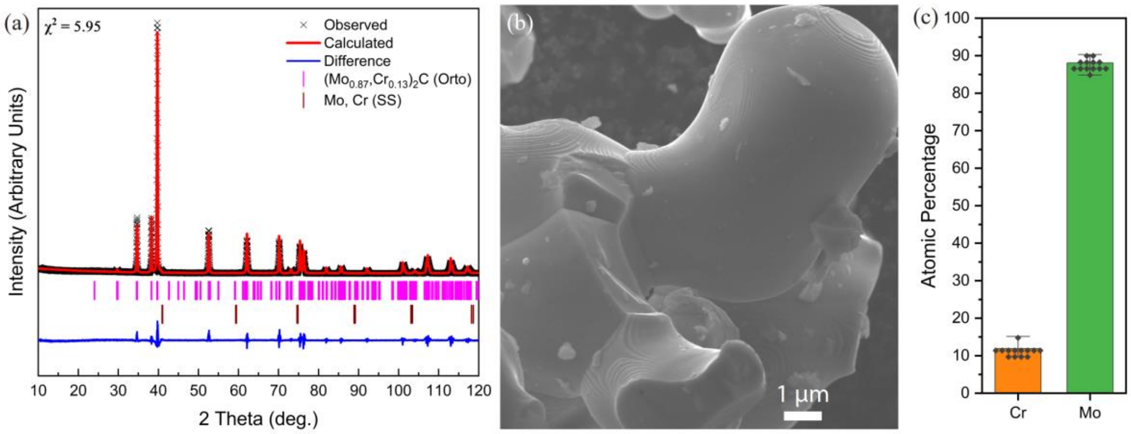

Figure S 1: Characterization of 3D $Mo_{2-x}Cr_xC$. (a) Rietveld analysis. The black 'X' represents the measured pattern, the red line is the Rietveld generated pattern and the difference between them is the blue line. The pink and brown ticks below the pattern represent the peak positions of the $Mo_{2-x}Cr_xC$ orthorhombic phase and Mo_xCr_{1-x} solid solution, respectively. (b) SEM micrographs showing the globular morphology with some laths on the edges. (c) Chemical composition obtained through EDX measurements on 15 individual particles.

Table S 1: Summary of Rietveld refinement of 3D $Mo_{2-x}Cr_xC$. The value in parentheses is the error (if not present, the parameter was not refined). The Cr occupancy was kept constant and was not refined (see Experimental Details).

Phases	$Mo_{2-x}Cr_x$	Mo_xCr_{1-x}
Space group	Pbcn, #60	Im-3m, #229
a (Å)	4.70176(3)	3.13675(17)
b (Å)	5.98448(5)	3.13675(17)
c (Å)	5.16726(4)	3.13675(17)
α	90.000	90.000
β	90.000	90.000
γ	90.000	90.000
Mo	8d, Occ = 6.96<br>0.2444(4) 0.1290(3) 0.0821(3)	2a, Occ = 1.74<br>0.0000 0.0000 0.0000
Cr	8d, Occ = 1.04<br>0.2444(4) 0.1290(3) 0.0821(3)	2a, Occ = 0.26<br>0.0000 0.0000 0.0000
C	4c, Occ = 4.00<br>0.0000 0.3640 0.2500	
Wt%	99.23 (1.05)	0.77 (0.22)
Rietveld Information	R_p: 20.7 R_{wp}: 25.8 R_{exp}: 8.62	Global Chi^2: 5.95

3D $Mo_{2-x}Cr_xGa_2C$ MAX PHASE

Table S 2: Summary of Rietveld refinement of $Mo_{2-x}Cr_xGa_2C$. The value in parentheses is the error (if not present, the parameter was not refined). The Cr occupancy was kept constant and was not refined (see Experimental Details).

Phases	$Mo_{2-x}Cr_xGa_2C$	$Mo_{2-x}Cr_xC$	$Mo_{2-x}Cr_xGaC$
Space group	P63/mmc, #194	Pbcn, #60	P63/mmc, #194
a (Å)	3.02684 (8)	4.70265(27)	3.02486(9)
b (Å)	3.02684 (8)	5.97835(62)	3.02486(9)
c (Å)	18.03214 (52)	5.18936(48)	13.10385(58)
α	90.000	90.000	90.000
β	90.000	90.000	90.000
γ	120.000	90.000	120.000
Mo	4f, Occ = 3.48<br>0.33333<br>0.66667<br>0.06489(16)	8d, Occ = 6.96<br>0.23078(156)<br>0.13334(164)<br>0.07634(255)	4f, Occ = 3.48<br>0.33333<br>0.66667<br>0.59009(20)
Cr	4f, Occ = 0.52<br>0.33333<br>0.66667<br>0.06489(16)	8d, Occ = 1.04<br>0.23078(156)<br>0.13334(164)<br>0.07634(255))	4f, Occ = 0.52<br>0.33333<br>0.66667<br>0.59009(20)
Ga	4f, Occ = 4.00<br>0.3333<br>0.66667<br>0.68279(16)		2c, Occ = 2.00<br>0.33333<br>0.66667<br>0.25000
C	2a, Occ = 2.00<br>0.0000<br>0.0000<br>0.0000	4c, Occ = 4.00<br>0.0000<br>0.3640<br>0.2500	2a, Occ = 2.00<br>0.0000<br>0.0000<br>0.0000
Wt%	42.34 (0.48)	9.13 (0.30)	48.53 (0.60)
Rietveld Information	R_p: 27.6 R_{wp}: 30.4	R_{exp}: 11.21	Global Chi^2: 8.70

Table S 3: Summary of global atomic percentages obtained from the high resolution XPS spectra.

	Mo at%	C at%	O at%	F at%
$Mo_{1.74}CT_z$	11.7 ± 0.4	68.5 ± 1.5	18.4 ± 0.5	1.4 ± 0.2

Table S 4: Comparison of chemical formulas fof various Mo-based MXenes: $Mo_{2-x}CT_z$ from $Mo_{2-x}Cr_xGa_2C$, $Mo_{4/3}CT_z$ from $(Mo_{2/3}Sc_{1/3})_2AlC$ and Mo_2CT_z from Mo_2Ga_2C.

MXene	Chemical formula from XPS	Total T_z	(O, OH):F ratio	Ref.
$Mo_{2-x}CT_z$	$Mo_{1.74\pm0.06}CO_{0.95\pm0.02}(OH)_{0.63\pm0.01}F_{0.3\pm0.03}.0.2\pm0.05H_2O_{ads}$ (base: C = 1)	1.88	1:0.2	This work
$Mo_{4/3}CT_z$	$Mo_{1.2\pm0.1}CO_{0.35\pm0.04}(OH)_{0.6\pm0.2}F_{1.2\pm0.2}.0.18\pm0.04H_2O_{ads}$ (base: C = 1)	2.15	1:1.3	[1]
Mo_2CT_z	$Mo_2C_{1.05\pm0.07}O_{0.7\pm0.1}(OH)_{0.9\pm0.2}F_{0.15\pm0.07}.0.5\pm0.2H_2O_{ads}$ (base: Mo = 2)	1.75	1:0.1	[1]

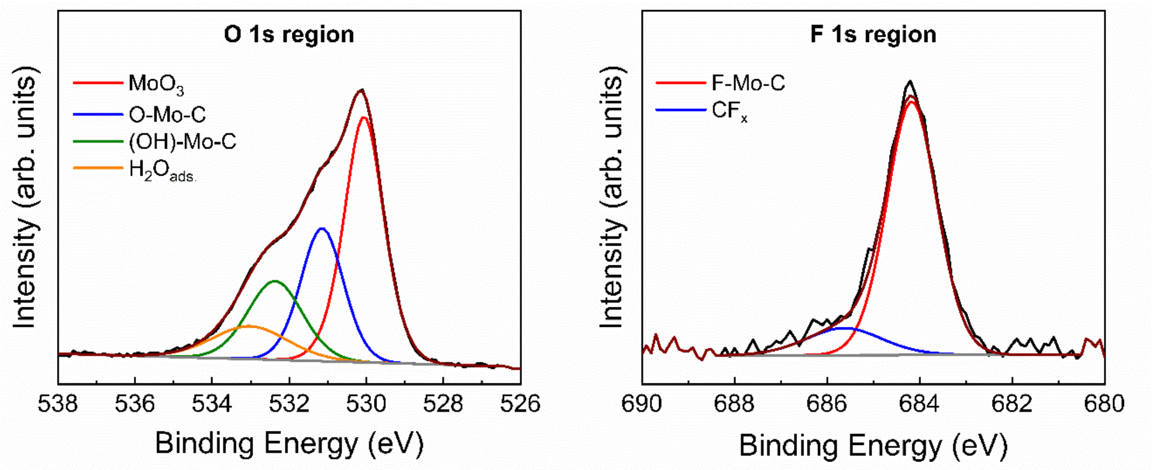

Figure S 2: XPS spectra of a $Mo_{2-x}CT_z$ film for the O 1s and F 1s regions. Colour coded peak fittings represent the assigned species tabulated in Table S 5.

Table S 5: XPS peak fitting results and their comparison to various Mo-based MXenes: $Mo_{2-x}CT_z$ from $Mo_{2-x}Cr_xGa_2C$, $Mo_{4/3}CT_z$ from $(Mo_{2/3}Sc_{1/3})_2AlC$ and Mo_2CT_z from Mo_2Ga_2C.

Region	Sample	BE [eV]	FWHM [eV]	Fraction	Assigned to	Ref
Mo 3d [a]	$Mo_{2-x}CT_z$	229.1 (232.3)	0.6 (0.8)	0.85	T_z-Mo-C	This work
		232.0 (235.1)	1.8 (1.8)	0.15	Mo^{+6}	232.6 (235.7) [2,3]
	$Mo_{4/3}CT_z$	229.2 (232.4)	0.6 (0.7)	0.78	T_z-Mo-C	[1]
		230.8 (233.8)	0.8 (1.6)	0.04	Mo^{+5}	231.2 (234.3) [2,3]
		232.0 (235.2)	1.3 (2.3)	0.18	Mo^{+6}	232.6 (235.7) [2,3]
	Mo_2CT_z	229.1 (232.3)	0.6 (0.9)	0.81	T_z-Mo-C	[1]
		230.6 (233.6)	0.5 (0.5)	0.01	Mo^{+5}	231.2 (234.3) [2,3]
		231.9 (235.1)	1.4 (1.7)	0.18	Mo^{+6}	232.6 (235.7) [2,3]
C 1s	$Mo_{2-x}CT_z$	282.9	0.8	0.09	T_z-Mo-C	This work
		284.4	1.7	0.03	C-C	[4–6]
		285.1	1.2	0.62	CH_x	[4–6]
		286.3	1.1	0.13	C-O	[7]
		287.4	2.2	0.13	C-F	[4–6]
	$Mo_{4/3}CT_z$	282.7	0.7	0.27	T_z-Mo-C	[1]
		284.8	1.3	0.43	C-C	[4–6]
		286.2	1.5	0.25	C-O	[4–6]
		287.6	1.3	0.04	C-F	[7]
		289.0	1.3	0.01	COO	[4–6]
	Mo_2CT_z	282.8	0.8	0.22	T_z-Mo-C	[1]
		284.6	1.3	0.42	C-C/CH_x	[4–6]
		285.9	1.5	0.32	C-O	[4–6]
		287.6	1.3	0.03	C-F	[7]
		288.8	1.3	0.01	COO	[4–6]
O 1s	$Mo_{2-x}CT_z$	530.1	1.2	0.45	Mo oxides	530.3 ± 0.4 eV [2,3]
		531.1	1.3	0.25	O-Mo-C	This work
		532.4	1.7	0.19	(OH)-Mo-C and/or OR[b]	This work
		533.0	2.3	0.11	H_2O_{ads} and/or OR[b]	This work
	$Mo_{4/3}CT_z$	529.8	1.1	0.21	Mo oxides	530.3 ± 0.4 eV [2,3]
		531.0	1.2	0.43	O-Mo-C	[8]
		532.3	1.5	0.20	(OH)-Mo-C and/or OR[b]	[8]
		533.2	1.9	0.16	H_2O_{ads} and/or OR[b]	[8]
	Mo_2CT_z	530.0	1.0	0.33	Mo oxides	530.3 ± 0.4 eV
		530.5	1.5	0.38	O-Mo-C	[9]
		531.8	1.7	0.17	(OH)-Mo-C and/or OR[b]	[9]
		533.0	2.0	0.12	H_2O_{ads} and/or OR[b]	[9]
F 1s	$Mo_{2-x}CT_z$	684.2	1.3	0.86	F-Mo-C	This work
		685.6	1.9	0.14	C-F	[7]
	$Mo_{4/3}CT_z$	684.0	1.2	0.79	F-Mo-C	[1]
		685.1	2.3	0.21	C-F	[7]
	Mo_2CT_z	684.2	1.5	0.82	F-Mo-C	[1]
		685.7	1.7	0.18	C-F	[7]

[a] Values in parenthesis correspond to $3d_{5/2}$ peaks. Areal ratios of $3d_{5/2}$ and $3d_{3/2}$ were constrained to 3:2.

[b] OR stands for organic compounds which are due to the exposure of the samples to the ambient and/or attached to MXene sheets during intercalation with TBAOH.

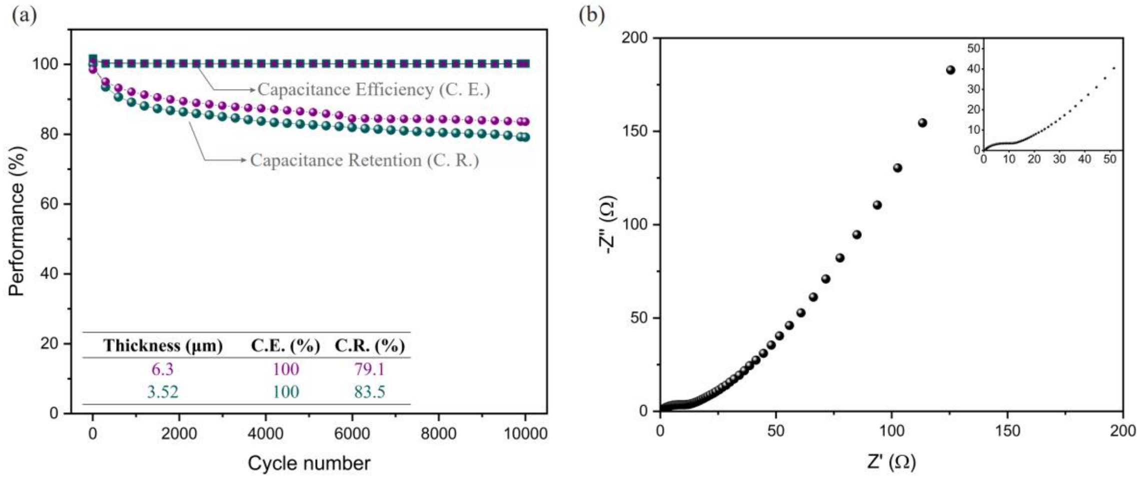

Figure S 3: (a) Stability plot, showing the relation of capacitance efficiency (C.E.) and retention (C.R.) with the number of cycles. The final numbers are showed in the table. (b) Nyquist plots of a vacuum filtered $Mo_{2-x}CT_z$ film (inset: high-frequency range).

REFERENCES

[1] W. Zheng, J. Halim, P. O. Å. Persson, J. Rosen, M. W. Barsoum, *J. Power Sources* **2022**, *525*, 231064.

[2] F. Werfel, E. Minni, *J. Phys. C Solid State Phys.* **1983**, *16*, 6091.

[3] K. Arata, M. Hino, *Appl. Catal.* **1990**, *59*, 197.

[4] A. Proctor, P. M. A. Sherwood, *Surf. Interface Anal.* **1982**, *4*, 212.

[5] Y. Xie, P. M. A. Sherwood, *Chem. Mater.* **1989**, *1*, 427.

[6] E. Desimoni, G. I. Casella, A. Morone, A. M. Salvi, *Surf. Interface Anal.* **1990**, *15*, 627.

[7] T. Nakajima, Y. Matsuo, B. žemva, A. Jesih, *Carbon N. Y.* **1996**, *34*, 1595.

[8] H. Lind, J. Halim, S. I. Simak, J. Rosen, *Phys. Rev. Mater.* **2017**, *1*, 044002.

[9] J. Halim, S. Kota, M. R. Lukatskaya, M. Naguib, M. Q. Zhao, E. J. Moon, J. Pitock, J. Nanda, S. J. May, Y. Gogotsi, M. W. Barsoum, *Adv. Funct. Mater.* **2016**, *26*, 3118.
